# Clinical validity of IntelliSpace Cognition digital assessment platform in mild cognitive impairment

**DOI:** 10.3389/fpsyg.2024.1451843

**Published:** 2024-12-30

**Authors:** Willem Huijbers, Nandor K. Pinter, Mandy Spaltman, Mike Cornelis, Ben Schmand, Baraa Alnaji, Maxwell Yargeau, Sarah Harlock, Ryu Platinum Dorn, Bela Ajtai, Erica S. Westphal, Gijs van Elswijk

**Affiliations:** ^1^Philips Research, Eindhoven, Netherlands; ^2^Dent Neurologic Institute, Amherst, NY, United States; ^3^Jacobs School of Medicine and Biomedical Sciences, University at Buffalo, Buffalo, NY, United States; ^4^Digital Cognitive Dx, Philips, Eindhoven, Netherlands; ^5^Faculty of Social and Behavioral Sciences, University of Amsterdam, Amsterdam, Netherlands

**Keywords:** digital health, Alzheimer's disease, neuropsychology, factor scores, cognitive composites

## Abstract

We evaluated a digital cognitive assessment platform, Philips IntelliSpace Cognition, in a case-control study of patients diagnosed with mild cognitive impairment (MCI) and cognitively normal (CN) older adults. Performance on individual neuropsychological tests, cognitive *z*-scores, and Alzheimer's disease (AD)-specific composite scores was compared between the CN and MCI groups. These groups were matched for age, sex, and education. Performance on all but two neuropsychological tests was worse in the MCI group. After ranking the cognitive scores by effect size, we found that the memory score was the most impaired, followed by executive functioning. The Early AD/MCI Alzheimer's Cognitive Composite (EMACC) and Preclinical Alzheimer's Cognitive Composite (PACC) scores were constructed from the digital tests on Philips IntelliSpace Cognition. Both AD-specific composite scores showed greater sensitivity and specificity than the Mini-Mental State Examination or individual cognitive *z*-scores. Together, these results demonstrate the diagnostic value of Philips IntelliSpace Cognition in patients with MCI.

## 1 Introduction

Digital cognitive assessments are increasingly used in patients with mild cognitive impairment (MCI) and Alzheimer's disease (AD) (Gold et al., 2018). The transition from traditional paper-and-pencil tests toward digital assessments is driven by a shortage of qualified neuropsychological staff and an increased demand from an aging population. Digital assessments also provide new opportunities to increase efficiency through automatic analysis, reduce human scoring errors, and quantify behavior in novel ways (Öhman et al., 2021). Akin to established paper-and-pencil tests, here we validate digitized cognitive tests in a clinical setting.

We evaluated a digital assessment platform, Philips IntelliSpace Cognition (ISC), in a case-control study of MCI patients. First, we assessed the performance of individual neuropsychological metrics from our digital cognitive battery and compared results with a group of cognitively normal older adults (CN) used in previous studies (Vermeent et al., 2022; Klaming et al., 2024). These studies demonstrated that the majority of these digitally assessed metrics are equivalent to their paper-and-pencil counterparts (Vermeent et al., 2022) and have moderate to excellent test-retest reliability (Klaming et al., 2024). Second, we evaluated a set of cognitive *z*-scores derived from a disease-agnostic factor model. This model was established in a larger healthy cohort (see Supplementary Table S1). So far, the digitized cognitive battery and the cognitive *z*-scores have not yet been validated in MCI patients. We expected to replicate findings of studies utilizing non-digital tools that found greater impairments in memory and executive functioning, while other cognitive domains, like visual spatial processing, are likely less impaired (Petersen et al., 1999; Cohen et al., 2022). Third, we identified two composite scores specific to Alzheimer's disease that can be constructed from a combination of outcome measures provided by the digital cognitive battery (Schneider and Goldberg, 2020; Jaeger et al., 2021). Disease-specific composites are used in clinical trials and often estimated by paper-and-pencil based tests. We have constructed the Early AD/MCI Alzheimer's Cognitive Composite (EMACC) (Jaeger et al., 2014) and the Preclinical Alzheimer's Cognitive Composite (PACC) (Donohue et al., 2014, 2017), from the digitally administered tests. Fourth, we compared the sensitivity and specificity of individual neuropsychological tests, the cognitive *z*-scores, and AD-specific composites. At the end, we provide a short comparison of our digital cognitive battery with other popular digital tools, in terms of diagnostic performance, required testing time, and available neuropsychological tests.

## 2 Materials and methods

### 2.1 Participants

This study was approved by the WCG Institutional Review Board Copernicus Group and the Internal Committee for Biomedical Experiments of Philips. The study is registered at clinicaltrials.gov (NCT04243642). Patients with a clinical diagnosis of mild cognitive impairment (MCI) were recruited from an outpatient neurology center (the Dent Neurologic Institute, Amherst, NY, USA). Eighty-one patients were included in the final analysis. All study participants signed an informed consent. The study inclusion criteria were based on Alzheimer's Disease Neuroimaging Initiative criteria (Petersen et al., 2010). Potentially eligible patients were identified through chart review from a pool of patients aged 50–90 years, with clinical diagnosis MCI or amnestic MCI, and confirmation of the diagnosis within the last 12 months. Patients who had an Mini Mental Status Exam (MMSE) score of not lower than 18 were included. The outcome of digital cognitive tests were not used to establish, exclude or reassign patients to the CN or MCI group, as the goal of our study was to evaluate the validity of the digital cognitive tests, and therefore, these should remain independent.

Patients were excluded if they had co-morbid neurological or psychiatric disorders known to affect cognition, vision impairment, hearing loss not corrected to normal, were currently admitted to a hospital, assisted living facility, nursing home, or psychiatric facility, had undergone a neuropsychological assessment in the past month, or had any of the following in their medical history: unconsciousness for more than 20 min related to traumatic brain injury or a head injury that resulted in an overnight hospital stay, any medical event requiring resuscitation in which they were unresponsive for more than 15 min, stroke, chemotherapy treatment within the past two months, current diagnosis or history of substance use or dependence, long-term alcohol abuse or daily alcohol consumption of more than four units, medical marijuana use or recreational marijuana use at least once per week, medications that may affect cognitive test performance (e.g., anticonvulsants, antipsychotics, benzodiazepines, opioids, tricyclic antidepressants, oxybutynin), or moderate to severe sleep apnea, defined as an apnea-hypopnea index equal to or >15. Patient eligibility was confirmed through existing medical records and the most recent clinical notes within 12 months of recruitment, and again during the study visit. Patients were also excluded if they had not had a Magnetic Resonance Imaging (MRI) exam of the brain in the preceding five years. MRI studies were reviewed by a neuroradiologist (NP). Patients were excluded if they had an MRI within the last 5 years that showed major structural abnormalities (including but not limited to brain tumor, encephalomalacia, lacunar infarct, hemorrhage, frontotemporal lobar degeneration, acute stroke, or cerebral amyloid angiopathy). Patients with a Fazekas score of 3 were excluded from the study.

The original chart review included 5,055 patient records, of which 4,589 were removed based on the above criteria. The remaining 466 patients were contacted for phone screening. Of the 466, 210 declined participation, 67 were not available via phone, 48 failed screening, 25 were scheduled for study visit but canceled, and four withdrew consent. Results of 29 MCI patients are not included due to an revision of the study protocol and software, as detailed later. Supplementary Table S2 shows a breakdown of screening and reasons for exclusion from patient population. In total, 81 MCI patients conducted the study according to final protocol. In the MCI patient group for 30.1% race/ethnicity was unknown or declined to answer, 64.2% self-identified as white, 3.7% as black/African-American, 1.2% as Asian and 2.4% as Latino-Hispanic. When we assume similar numbers for the patients with missing responses, 92.9% would self-identify as white, 5.3% as black/African-American, 1.2% as Asian and 2.4% as Latino-Hispanic. The other demographics are in Table 1.

**Table 1:** Descriptive statistics.

		**CN**	**MCI**	***p*-value**	**Effect**	**Used in**	**Used in**	**Used in**
		***(N = 81)***	***(N = 81)***	**bonferroni**	**size**	**CZS**	**EMACC**	**PACC**
Age (years)		73.3 ± 6.75	74.1 ± 7.65	1.000	−0.12			
Sex (n)	Female	38 (47%)	40 (49%)	1.000	0.05			
	Male	43 (53%)	41 (51%)					
Education (years)		17.6 ± 2.92	17.8 ± 3.08	1.000	−0.11			
RAVLT (total score)	Learning trials	40.9 ± 7.52	30.0 ± 9.91	< 0.001	1.25	×	×	–
	Immediate recall	7.40 ± 2.98	3.85 ± 3.21	< 0.001	1.15	×	–	-
	Delayed recall	7.70 ± 3.08	4.47 ± 3.41	< 0.001	1.00	×	–	×
Trail making (duration)	Test A	44.0 ± 12.4	52.8 ± 28.4	0.221	−0.40	×	×	–
	Test B	113 ± 46.3	176 ± 131	0.002	−0.65	×	×	–
Clock test (total score)	Drawing	18.6 ± 2.45	18.0 ± 3.83	1.000	0.20	–	–	–
	Copy	19.5 ± 1.84	18.6 ± 3.93	1.000	0.28	–	–	–
Star Cancellation (duration*)		0.92 ± 0.25	1.16 ± 0.50	0.004	−0.60	×	–	–
ROCFT (total score)	Copy	30.0 ± 5.47	28.5 ± 7.28	1.000	0.23	×	–	–
	Immediate recall	9.58 ± 5.78	6.09 ± 5.51	0.002	0.62	×	–	–
Digit span (total score)	Forward	7.84 ± 2.11	6.35 ± 2.21	< 0.001	0.70	×	-	–
	Backward	6.51 ± 2.66	5.35 ± 2.33	0.065	0.47	×	-	–
Letter fluency (total score)		39.7 ± 12.2	36.0 ± 11.3	0.810	0.32	×	–	–
Category Fluency (total score)	Trial animals	20.7 ± 5.58	15.4 ± 5.13	< 0.001	1.00	–	×	×
	Trial vegetables	12.4 ± 2.99	10.1 ± 4.00	0.004	0.63	–	–	–
	Trial fruit and furniture	12.6 ± 2.99	9.06 ± 3.19	< 0.001	1.19	–	×	×
SNMT (total score)		37.4 ± 3.99	34.2 ± 8.56	0.396	0.46	–	×	×
MMSE (total score)		27.9 ± 1.42	26.4 ± 2.68	0.001	0.68	–	–	×
Memory	*z*-score	0.10 ± 0.85	−1.07 ± 1.04	< 0.001	1.24			
Executive functioning	*z*-score	0.00 ± 0.80	−0.87 ± 0.95	< 0.001	1.00			
Working memory	*z*-score	0.04 ± 0.80	−0.63 ± 0.72	< 0.001	0.88			
Visual spatial processing	*z*-score	0.20 ± 0.68	−0.50 ± 0.95	< 0.001	0.84			
Processing speed	*z*-score	−0.07 ± 0.91	−0.71 ± 1.18	0.001	0.61			
Verbal processing	*z*-score	−0.09 ± 1.07	−0.46 ± 1.05	0.168	0.35			

CN, cognitively normal; MCI, mild cognitive impairment; Effect Size is quantified by Cohen's *d*, the *p*-values reflect a two-sample *t*-test, or chi-square test, corrected for multiple comparisons; RAVLT, Rey's Auditory Verbal Learning Test; duration*, duration per correct cancellation in seconds; ROCFT, Rey-Osterrieth Complex Figure Test; SNMT, Symbol Number Matching Test; MMSE, Mini Mental State Examination version 2; The SNMT has a smaller number of observations for CN (*n* = 22). In the three columns on the right, the X marks indicate what neuropsychological tests/metrics are used for the Cognitive *Z*-scores (CZS), Early/Mild Alzheimer's Cognitive Composite (EMACC) and Pre-clinical Alzheimer's Disease Cognitive Composite (PACC).

The control group consisted of 81 cognitively normal adults selected from a larger U.S. reference population. See statistical methods for details on selection procedure. The total U.S. reference cohort consists of 687 healthy participants, representative of the U.S. census with regard to age, sex, education, and race/ethnicity. These data were collected in two separate studies between 2019 and 2021 (NCT0380138/NCT04729257) in four states across the U.S., including New York, Pennsylvania, Florida, and California. We ensured that the control group only included cognitively normal (CN) older adults, applying the Alzheimer's Disease Neuroimaging Initiative (ADNI) criteria for healthy controls (Petersen et al., 2010). After selection, the control group was matched to the MCI cohort. These matched CN older adults self-identified for 87.7% as white, 7.4% as black/African-American, 2% as Asian and 4% as Latino-Hispanic (see Supplementary Table S1). After selection, the control group was no longer representative for the U.S. population, but matched the demographics of MCI patients at Dent Neurologic Institute in terms of age, sex and education.

### 2.2 Cognitive battery

The cognitive battery was administered in-person on an iPad Pro tablet (screen size of 12.9 inches and a 2,732 × 2,048 resolution), using the Philips IntelliSpace Cognition application, which is approved by the US Food and Drug Administration as a Class II medical device (GUDID 00884838108554). Instructions and guidance were provided to the participants in both written and verbal form. The participants used verbal responses, touch of the keypad and drawing with a digital pencil or finger to perform the tests.

The research personnel were individually trained on the use of Philips IntelliSpace Cognition. Research personnel was always present during administration of the cognitive tests to ensure that the patients understood and followed instructions and to administer questionnaires and paper-and-pencil screeners. Research personal was instructed to provide minimal assistance with the cognitive tests. Most of the patients performed the tests independently, requiring very little supervision from the research personnel. The most frequently used guidance was prompting the patient to replay the test instructions. Each participant completed the assessment.

The predefined cognitive battery included Rey's Auditory Verbal Learning Test (RAVLT), Trail-Making Test (TMT) A and B, Star Cancellation Test, Rey-Osterrieth Complex Figure Test (ROCFT), Letter Fluency also known as the Controlled Word Association Test (COWAT), Digit Span Forward and Backward, and four additional cognitive tests: Clock Test Drawing and Copy, Category Fluency and the Symbol Number Matching Test (SNMT), also known as, or equivalent to, the Symbol Digit Modalities Test or Digit Symbol Substitution Test (Strauss et al., 2006). Digital versions of the Mini-Mental State Exam version 2, (MMSE) (Folstein et al., 1975), the Patient Health Questionnaire (PHQ-9) and General Anxiety Disorder-7 (GAD-7) were also administered. Only the MMSE, but not the two questionnaires are included in the analyses. The verbal responses were automatically transcribed by voice recognition software and the drawing tests were analyzed by proprietary computer vision algorithms. These automated annotation algorithms have been validated against human expert raters (Vermeent et al., 2022, 2021). For participants who were unable to reach the last target on TMT (either A or B), and thus did not complete the test, the normed score was imputed based on the lowest norm percentile. This is in line with the TMT manual and prevents misrepresentation of a shorter duration due to participants not finishing the TMT. In the MCI group, this occurred three times for TMT-A and 12 times for TMT-B. In the CN group, this occurred twice for TMT-B.

### 2.3 Cognitive *z*-scores

The predefined cognitive battery was used to estimate the cognitive *z*-score (see Table 1). The *z*-scores could not be defined on a certain predefined and additional cognitive tests (Clock Test, Category Fluency and SNMT) because these additional tests were not available in the US cohort study, and could therefore not be corrected for age, sex and education. First, we used a confirmatory factor model to estimate a disease-agnostic profile in six cognitive domains: memory, executive functioning, processing speed, verbal processing, visual spatial processing and working memory. The factor loading's for these domains were derived from a structural equation model that was similar (but not identical) to models we have used in previous work on the healthy individuals (Vermeent et al., 2022, 2020). The Supplemental material include the predefined structure of the confirmatory factor model. Processing speed and Verbal Processing are defined by a single cognitive test. Supplementary Figure S1 shows the demographics of the U.S. cohort that was used to establish these factor weights. Supplementary Table S1 shows the factor weights for each cognitive *z*-score. Note that consistent with the predefined structure, processing speed and verbal processing only load upon a single neuropsychological test. Next, before the factor weights were applied, each neuropsychological test was transformed to a *z*-score and corrected for age, sex and education. Finally, the established factor weights were applied to derive the cognitive *z*-scores. Because the factor weights sum to one, the results are also standard scores, but adjusted for age, sex, and education.

### 2.4 Disease-specific composites

We evaluated two disease-specific composite scores: the Early AD/MCI Alzheimer's Cognitive Composite (EMACC) and the Preclinical Alzheimer's Cognitive Composite (PACC). EMACC was specifically designed as a cognitive endpoint for clinical trials of early AD (Jaeger et al., 2014). It includes neuropsychological tests from the domains of memory, executive functioning, and processing speed. The exact neuropsychological tests, or versions of them, used to construct the EMACC vary slightly between studies (Jaeger et al., 2014, 2021). In this study, EMACC was defined similar to the Mayo Clinic Study of Aging, using the RAVLT learning total score, category fluency-animals and category fluency fruit and Furniture, TMT-A, TMT-B, and SNMT.

The PACC is designed for the detection of subtle cognitive changes in the early stages of the disease continuum (Donohue et al., 2014, 2017), and includes neuropsychological tests from the domains of memory and executive functioning, as well as a measure of general cognition. In this study, the PACC was defined similar to the extended definition using a measure of semantic processing (Papp et al., 2017). As in the EMACC, the exact tests used to construct the composite score vary slightly between various studies and clinical trials (Donohue et al., 2014; Petersen et al., 2010; Fowler et al., 2021; Dagley et al., 2017; Papp et al., 2017; Jessen et al., 2022). Instead of the free and cued selective reminding test (FCSRT), we used the RAVLT delayed recall, akin to the PACC definitions that use the California Verbal Learning Test delayed recall (Donohue et al., 2014; Fowler et al., 2021). Table 1 lists the neuropsychological tests/scores used for the EMACC and PACC. Both EMACC and PACC use *z*-scoring to normalize the test scores in order to combine them into a single composite score. We estimate the *z*-scores using the same normative cohort used for the confirmatory factor analysis. Note that the *z*-transform does not correct the disease-specific composites for age, sex, or education.

### 2.5 Statistics

We planned comparisons between MCI patients and a matched healthy control group using independent samples *t*-tests, chi-squares tests and logistic regression models. Additionally, we aimed to assess the accuracy, sensitivity, and specificity of the highest-ranking cognitive *z*-scores and disease-specific composite scores. Statistical analyses were performed using R v4.1.1 and the base R stats package (R Core Team, 2021). Figures and tables were generated with ggplot2 v3.3.3 (Wickham, 2016) and the table1 package v1.4.2 (Rich, 2023). Receiver operating characteristic (ROC) curves were calculated using the pROC package v1.18.0 (Robin et al., 2011) and statistically compared using bootstrap tests for two correlated ROC curves (*n* = 2,000). Evaluation metrics for sensitivity, specificity, and area under the curve (AUC) were calculated using the cutpointr package v1.1.1 (Thiele and Hirschfeld, 2021), with the optimal point on the ROC curve determined by the product of sensitivity and specificity.

The control group of cognitively normal (CN) older adults was selected with a non-parametric matching procedure (Ho et al., 2007) from a normative cohort (see Supplemental Figure S1; Vermeent et al., 2020, 2022). First, we removed all CNs with an MMSE < 25 and RAVLT immediate recall score one standard deviation below the norm, similar to ADNI criteria for healthy controls (Petersen et al., 2010). Next, we employed nearest-neighbor matching based on a propensity score, as implemented in the MatchIt package v 4.3.2 (Ho et al., 2011). The propensity score was estimated using a logistic regression model with age, sex, and education level. Table 1 shows the descriptive statistics that compare the CNs matched with the MCI patients. The *p*−values are based on two-sample *t*-tests, or in the case of sex, a chi-square test. The matching procedure ensures that comparisons of various tests, cognitive *z*-scores, and disease-specific composites between CN and MCI are not simply driven by demographic factors.

## 3 Results

### 3.1 Descriptive results

The group of MCI patients performed significantly worse on all tests (see Table 1), with the exception of the ROCFT Copy and the Clock Drawing Test, where the performance differences were not significant. Based on the effect size, quantified by Cohen's *d*, the RAVLT Learning Trials showed the largest difference between CN and MCI, and similar effects were observed with RAVLT Immediate Recall, Delayed Recall, and Category Fluency. The effect size was more modest for the other tests. Also, for most neuropsychological tests, and the cognitive score, the standard deviation was larger in the MCI group (see Table 1).

**Figure 1:**
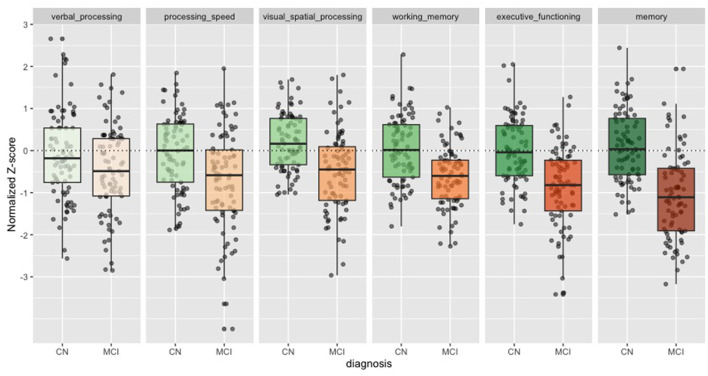
Cognitive *z*-scores in CN and MCI. Boxplots for each of the cognitive *z*-scores. Cognitively Normal (CN) in green. Mild Cognitive Impairment (MCI) in orange. Dots show individual values.

The cognitive *z*-scores were ranked by their relative effect size in Table 1. The memory score showed the largest effect size (Cohen's *d* = 1.24), followed by executive functioning (Cohen's *d* = 1.00), working memory (Cohen's *d* = 0.88), and visual spatial processing (Cohen's *d* = 0.84)—all greater than MMSE. Processing speed (Cohen's *d* = 0.61) and Verbal processing showed the smallest effect sizes (Cohen's *d* = 0.35). In Figure 1 we show the individuals *z*-scores for CN and MCI. These *z*-scores are corrected for age, sex and education.

### 3.2 Sensitivity and specificity

**Figure 2:**
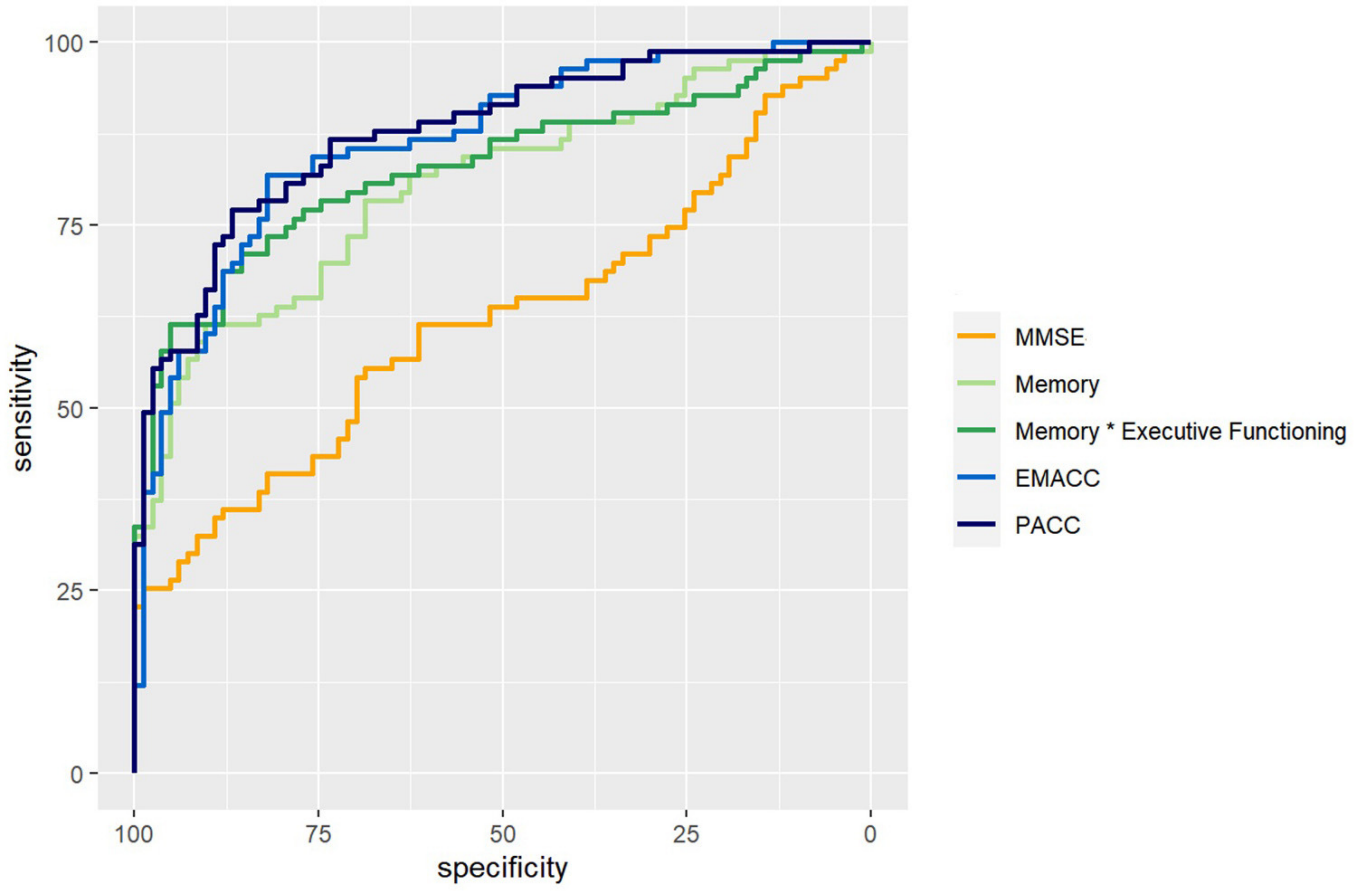
ROC curves. ROC curves for logistical regression models that classify Cognitively Normal (CN) vs. Mild Cognitive Impairment (MCI). In orange, a model based on the Mini Mental State Exam (MMSE). In light green, based on the memory score. In dark green, memory by executive functioning. In light blue, the Early/Mild Alzheimer's Cognitive Composite (EMACC), and in dark blue the Pre-clinical Alzheimer's Disease Cognitive Composite (PACC).

We used logistic regression models to classify the diagnosis of CN versus MCI based on demographic factors, MMSE, cognitive *z*-scores, and disease-specific composites. We calculated sensitivity, specificity, and area under the curve (AUC) as shown in Table 2. We also visualized the ROC curves for a selection of models (Figure 2). The prevalence estimate (and majority baseline) is 0.5 since the CN and MCI groups are balanced. The AUC of models that use age, sex, and education is at the chance level as a consequence of the matching procedure. Consistent with the largest effect size (Table 1), we found that *z*-scores for memory and executive functioning were the strongest predictors of the diagnosis. We also examined whether a combination of cognitive *z*-scores would improve classification by running logistic regression models for all possible combinations of two domain scores, including an interaction term. The logistic regression model with memory * executive functioning performed best and is included in Table 2. However, it only marginally improved AUC relative to a model with only memory (see also statistical comparisons below). The model that used the PACC showed the highest AUC, closely followed by the EMACC.

**Table 2:** Sensitivity, specificity, and AUC.

**Predictor**	**Sensitivity**	**Specificity**	**AUC**	**CI 95%**
PACC	0.84	0.79	0.87	0.81–0.93
EMACC	0.79	0.81	0.87	0.81–0.92
Memory * Executive functioning	0.86	0.70	0.84	0.77–0.91
Memory	0.88	0.64	0.81	0.74–0.87
Executive functioning	0.79	0.60	0.75	0.68–0.82
Working memory	0.62	0.74	0.73	0.65–0.80
Visual spatial processing	0.67	0.69	0.72	0.64–0.80
Processing speed	0.64	0.62	0.66	0.58-0.84
MMSE (reference)	0.67	0.57	0.65	
Verbal Processing	0.64	0.53	0.59	0.52–0.66
Age	0.74	0.38	0.54	0.45–0.63
Education	0.54	0.54	0.53	0.42–0.64
Sex	0.47	0.51	0.49	0.38–0.60

Table with sensitivity, specificity, Area Under the Curve (AUC) and Confidence Interval (CI) for logistical regression models that classify CN vs. MCI. The model predictors are shown in the first column.

PACC, preclinical Alzheimer's cognitive composite; EMACC, early AD/MCI Alzheimer's cognitive composite; MMSE, Mini Mental State Examination; Memory * Executive Functioning indicates a model with both cognitive *z*-scores and the interaction term.

The results of pairwise statistical comparisons for a selection of ROC curves can be seen in Figure 2. First, we compared the ROC curves with the ROC curve for MMSE, Memory > MMSE (*D* = 3.36; *p* < 0.001), Memory * Executive Functioning > MMSE (*D* = 4.32; *p* < 0.001), EMACC > MMSE (*D* = 4.95; *p* < 0.001) and PACC > MMSE (*D* = 5.98; *p* < 0.001). In the comparisons with the Memory *z*-score, we found smaller differences: Memory * Executive Functioning > Memory (*D* = 1.86; *p* = 0.032), EMACC > Memory (*D* = 1.89; *p* = 0.029), PACC > Memory (*D* = 2.21; *p*=0.014). When comparing the disease-specific cognitive composites to Memory * Executive Functioning, we found no statistical differences: EMACC > Memory * Executive Functioning (*D* = 1.06; *p* = 0.145) and PACC > Memory * Executive Functioning (*D* = 1.31; *p* = 0.096). Similarly, we did not find any evidence for a difference between the PACC > EMACC (*D* = 0.378; *p* = 0.353).

### 3.3 Comparison with other digital cognitive tools

Here, we provide a short comparison of our digital cognitive battery with other digital assessment tools, in terms of diagnostic performance, required testing time, and available neuropsychological tests. Together these metric provide an indication of the potential impact on clinical workflow. Below, we compared our tool with the Brain Health Assessment-Cognitive Score (BHA-CS), the CogState Brief Battery, BrainCheck, the Computer-Administered Neuropsychological Screen for Mild Cognitive Impairment (CANS-MCI), and Cambridge Neuropsychological Test Automated Battery Paired Associates Learning (CANTAB-PAL).

The CogState Brief Battery generates two composite scores: psychomotor/attention and learning/working memory. Sensitivity and specificity were 41 and 85% for the former, and 80 and 86% for the latter. The CogState Brief Battery uses four tests to measure episodic memory, working memory, attention, and processing speed, in a self-administered, 15-min-long battery (Maruff et al., 2013). The Brain Health Assessment-Cognitive Score has shown the same sensitivity as IntelliSpace Cognition (ISC) but higher, 85% specificity, in a study of 451 cognitively normal subjects, 289 patients with MCI and 110 patients with mild dementia. This assessment is 20 min long and provides information on four of the six cognitive domains measured by the ISC battery, plus category fluency (animals) for language (Tsoy et al., 2020). The memory domain is based on associative memory. The BrainCheck computerized battery showed a sensitivity of 86% and a specificity of 83% to classify between MCI patients and normal controls (AUC = 0.84) in a recent study of 35 cognitively normal subjects, 22 MCI, and 42 dementia patients (Ye et al., 2022). This battery was based on five tests, duration was not reported in the study, but on the company website they report 20 min. The highest sensitivity was found with the CANS-MCI screening tool, which had 89% sensitivity while only 73 specificity (Ahmed et al., 2012); however, this study had a small sample size, with 15 MCI cases and 20 controls. CANS-MCI is 30 min long and assesses two cognitive domains (episodic memory and executive functioning) of the six domains measured by the ISC battery. CANTAB-PAL, a standalone test of episodic memory, showed similar sensitivity and better specificity than ISC, in a study of 30 healthy elderly and 17 MCI patients (Barnett et al., 2016).

When comparing these assessments by the cognitive domains or functions they measure (Table 3), the ISC battery provides a relatively comprehensive assessment, which, in turn, leads to a longer examination time. All listed tools assess episodic memory, although the BHA-CS measures associative a memory, a contributor component of episodic memories. The next most commonly measured domain is executive functioning, which is included in the BHA-CS, BrainCheck, and CANS-MCI, but is absent from both the CANTAB-PAL and CogState Brief Battery. This is followed by attention, which is included in CogState Brief Battery and BrainCheck—it is not a separate domain in the cognitive model of ISC but embedded in different domains. Similarly, visual spatial processing is included only in BHA-CS, and BrainCheck, although the latter specifies spatial awareness. Processing speed is measured with BA-CS and CogState Brief Battery, and working memory is measured with CogState Brief Battery. Letter fluency is only found in ISC, and defines the verbal processing *z*-score. The BHA and CANS-MCI also measure language functions, with category fluency and picture naming tests. The category fluency tests were also obtained in the current study with ISC, but are not used by the factor model. In summary, ISC compares favorably with these previously reported tools when comparing sensitivity and specificity. Yet, ISC also stands out with its test duration, which is 1.5–2.5 times longer. Below we provide an interpretation of these results.

**Table 3:** Category Fluency is not part of the model.

	**Episodic**	**Executive**	**Attention**	**Visual spatial**	**Processing**	**Working**	**Verbal**	**Language**
	**memory**	**functioning**		**processing**	**speed**	**memory**	**processing**	
IntelliSpace Cognition	X	X	X	X	X	X	X	Category fluency
Cogstate brief battery	X	–	X	–	X	X	–	–
Braincheck	X	X	X	Spatial awareness	–	–	–	–
Brain health assessment	Associative	X	–	X	X	–	–	Category fluency
CANS-MCI	X	X	–	–	–	- -	–	Picture naming
CANTAB_*a*_	X	X	X	–	X	X	–	–

IntelliSpace Cognition (ISC) includes a confirmatory factor model, that provides cognitive *z*-scores in several domains. In the current factor model, Processing Speed and Verbal Processing are based on a single normalized cognitive test. Category Fluency is not part of the model, but can be used as an individual test of Language. The other digital assessment tools do not include a pre-specified factor model, but include cognitive tests from these cognitive domains. CANTAB_*a*_ also has tests in these domains, in the discussion we specifically discuss CANTAB-PAL.

## 4 Discussion

Our study demonstrates the clinical validity of a digital cognitive platform, IntelliSpace Cognition, in patients with MCI. We found that performance in MCI patients was significantly worse compared to cognitively normal adults, on almost all neuropsychological tests. Furthermore, performance on some tests was impacted more than on others. Specifically, from the six cognitive scores investigated, the memory score was most abnormal. Finally, AD-specific composite scores showed greater sensitivity and specificity than the cognitive *z*-scores, individual neuropsychological tests, and the MMSE.

When comparing the performance of individual neuropsychological tests between CN and MCI (see Table 1), RAVLT, a test of learning and memory, was most affected in MCI, consistent with diagnostic criteria (Petersen et al., 1999). Category fluency showed the second largest effect size, while letter fluency was much less impacted. This replicates previous findings (Rinehardt et al., 2014; Mirandez et al., 2017; Balthazar et al., 2007). In general, the cognitive *z*-score reflects these individual test results: memory showed the greatest difference in the MCI group, which follows from the factor model dependencies of this domain on the RAVLT(Vermeent et al., 2022). Executive functioning score showed the second most difference between CN and MCI; a finding that also replicates previous work (Crowell et al., 2002; Guarino et al., 2020; Schmitter-Edgecombe et al., 2009). The cognitive *z*-score for verbal processing was only based on letter fluency, not category fluency. This explains the small difference between CN and MCI for verbal processing. A different factor model, that would have included category fluency, would likely have found a larger differences. The logistical regression that included memory by executive functioning showed the highest AUC except for the PACC and EMACC (see Table 2). This model slightly outperformed a logistic classifier that used only the memory *z*-score. This result is consistent with current perspectives on the importance of measuring multiple cognitive domains for the diagnosis of MCI or mild dementia (Schneider and Goldberg, 2020; Weintraub et al., 2018).

We constructed two digital cognitive composite scores: the EMACC and PACC (Donohue et al., 2014; Jaeger et al., 2014). The EMACC and PACC classified CN versus MCI patients with an AUC of 0.87 and 0.88 respectively (Table 2). Both AD-specific composites outperform the MMSE, individual neuropsychological tests, and the cognitive *z*-scores. The logistic regression model that included both memory, executive functioning was not statically different from the EMACC and PACC. These findings are consistent with the notion that MCI should not be defined by an impairment in a single cognitive domain. It is worth noting that the PACC includes the MMSE, as a measure of global cognition; in contrast, the EMACC does not. Our findings do not elucidate if a measure of global cognition improves detection, as the difference between PACC and EMACC was also not significant. Both composites were originally designed to quantify early cognitive impairment. Therefore, it is no surprise that they are sensitive to differences between CN and MCI patients. Importantly, these composites were originally designed with conventional paper-and-pencil neuropsychological tests. Here, we replicate this work, but using a digital cognitive assessment platform.

We also compared our results with published reports from other digital assessment tools. However, without a head-to-head study, a direct comparison between tools remains challenging. As each tool has been evaluated in a different cohort, with various inclusion/exclusion criteria. Also, in many other studies, patients are diagnosed, or (re)classified as CN, MCI or dementia, after neuropsychological testing (Ellis et al., 2009; Bondi et al., 2014). Although this is consistent with the clinical use of neuropsychological tests, it results in an overestimation of the accuracy, as compared to clinical practice. In our study, we did not reclassify patients after neuropsychological tests. Thus, these results probably underestimate the diagnostic performance of IntelliSpace Cognition, relative to several of the other digital assessment tools.

In summary, with regard to sensitivity and specificity of classifying MCI, Intellispace Cognition (ISC) compares favorably with previously reported tools. Yet, ISC also stands out with a considerately longer test duration. However, the longer testing time yields proportionally more data points, as shown in (Table 3) allowing ISC to provide an assessment that is closer to a standard neuropsychological evaluation in breadth, while the other tools have a narrower functionality. The implications of this will be discussed below under Future Directions.

### 4.1 Limitations

This was a single-center study with a modest sample size of 81 MCI patients. The clinical diagnosis was based on MMSE scores and the clinical opinion of a small number of providers, thus differences could occur if compared to other practices in a multi center study. Secondly, the MCI patients recruited at our memory clinic where relatively highly educated (17.8 ± 3.08), with the majority holding a masters degree or equivalent. This might be caused by a more general bias, as dementia is under diagnosed in persons who have lower educational attainment and in racial/ethnic minorities (Amjad et al., 2018). Although, the differences between CN and MCI cannot be explained by education, due to our matching-procedure, this limits the external validity of our results. Third, traditional paper-and-pencil tests were not administered in MCI patients, thus we lack a direct comparison between an interpretation by a neuropsychologist and the automated ISC score and cannot determine if the sensitivity or specificity of our digital cognitive platform is smaller or greater than traditionally administered tests with manual scoring. However, test scores are similar to scores reported for traditional paper-and-pencil tests in MCI, and the equivalence of IntelliSpace Cognition to paper-and-pencil tests with human raters have previously been demonstrated in CN adults (Vermeent et al., 2022). Thus, although we cannot exclude minor differences, we can infer that the platform performs on-par with traditional paper-and-pencil tests. Fourth, no amyloid-β or tau biomarkers were obtained. Therefore, we cannot characterize these patients in terms of AD research stages (Sperling et al., 2011; Dubois et al., 2016). The inclusion criteria were based on current clinical guidelines and included MRI. The resulting MCI group was probably heterogeneous in terms of pathological changes, consisting of patients with and without amyloid, tau and some degree of vascular pathology. Nevertheless, recent biomarker-based studies reported similar results when using the PACC composite score (Ding et al., 2022; Öhman et al., 2021).

### 4.2 Future directions

Future validation work should address the limitations of our study, by including larger sample sizes, other composite scores that include functional outcome metrics, as well as cohorts of prospectively enrolled patients. Comparisons with blood or fluid biomarkers and neuroimaging should also be conducted. The role of ISC in clinical practice remains to be determined: a shorter test may suffice as a screening tool, but for monitoring clinical progression, a more detailed cognitive assessment is valuable. Additionally, recent research suggests that cognitively normal adults with positive amyloid and tau biomarker status are likely to experience cognitive decline (Ossenkoppele et al., 2022). However, many patients do not fit this profile, and the boundaries between positive and negative biomarker statuses are often not clear-cut. For instance, patients harboring only tau also exhibit impairments in different cognitive domains (Quintas-Neves et al., 2022). Therefore, cognitive measurements will likely continue to enhance clinical forecasting. An accurate assessment of cognition, along with the biological characterization of AD, is crucial in making decisions about starting or stopping a treatment. Digital tools have advantages over traditional paper-and-pencil methods for operational reasons and are likely to play a key role in treatment decisions and monitoring treatment efficacy (Assunção et al., 2022).

In conclusion, the IntelliSpace Cognition platform is a promising tool to characterize cognitive deficits in patients with MCI. Compared to most other digital assessment tools, the IntelliSpace Cognition battery is more comprehensive, but also longer. Further research should focus on other on larger cohorts for validation, as well as combining cognitive data with Alzheimer's disease biomarkers.

## Data Availability

The raw data supporting the conclusions of this article will be made available by the authors, without undue reservation.
