# Supplementary material for: Clinical validity of IntelliSpace Cognition digital assessment platform in mild cognitive impairment
Source: Front Psychol. 2024 Dec 30;15:1451843. doi: 10.3389/fpsyg.2024.1451843 (PMC11726315; doi:10.3389/fpsyg.2024.1451843)
Supplement: Supplementary file 1 [file Data_Sheet_1.PDF]

frontiers

Supplementary Material

Table S1. Demographics US cohort

		matched CN (N=81)	non-matched CN (N=606)	Overall US (N=687)
**Age (years)**		73.26 (6.75)	47.53 (16.37)	50.56 (17.62)
**Sex)**				
	Female	38 (46.9%)	336 (55.4%)	374 (54.4%)
	Male	43 (53.1%)	270 (44.6%)	313 (45.6%)
**Education (years)**		17.60 (2.92)	16.54 (2.86)	16.67 (2.89)
**Race)**				
	American Indian/Alaska Native	0 (0.0%)	1 (0.2%)	1 (0.1%)
	Asian	2 (2.5%)	31 (5.1%)	33 (4.8%)
	Asian/White	0 (0.0%)	1 (0.2%)	1 (0.1%)
	Black/African-American	6 (7.4%)	84 (13.9%)	90 (13.1%)
	Black/African-American/White	0 (0.0%)	2 (0.3%)	2 (0.2%)
	White	71 (87.7%)	451 (74.4%)	552 (76.0%)
	Native Hawaiian/Other Pacific Islander	0 (0.0%)	5 (0.8%)	5 (0.7%)
	None or Other	2 (2.5%)	31 (5.1%)	33 (4.8%)
**Ethnicity)**				
	Hispanic/Latino	3 (3.7%)	89 (14.7%)	92 (13.4%)
	No Hispanic/Latino	87 (96.3%)	517 (85.3%)	595 (86.6%)

Table Legend: Demographics of the entire US cohort. First column shows the demographics for the Cognitively Normal (CN) adults that have been matched to the patient cohort. The second column the non-matched CN, and the last column the overall US cohort.

Table S2. Recruitment

	N	Percentage (%)
**Total Number of Reviewed Charts**	**5055**	
**Excluded**	**4589**	**90.8**
Progressed to Dementia	865	17.1
Lost to Follow Up	691	13.7
Ineligible Medication	644	12.7
Stroke	378	7.5
>90 years old	286	5.7
Other Neurodegenerative Disease	266	5.3
Abnormal MRI	254	5
Moderate-Severe Sleep Apnea	239	4.7
Deceased	211	4.2
<50 years old	178	3.5
Severe Mood or Psychiatric Disorder	166	3.3
Other (e.g. aphasia, learning disability)	144	2.8
TBI, ECT, CNS Radiation, Brain surgery	81	1.6
Substance abuse	75	1.5
Medical Cannabis	73	1.4
Epilepsy	38	0.8
**Eligible for Recruitment**	**466**	**9.2**
Declined prior to Phone Screening	181	38.9
Unable to Reach	67	14.4
Did not pass Phone Screening	48	10.3
Declined after Phone Screening	29	6.2
Canceled or No-show	25	5.4
Withdrawn Consent	4	0.9
Completed a Study Visit	112	24.0
**Completed final protocol**	**81**	**17.4**

Breakdown of screening for MCI patients

Figure S1. Weights confirmatory factor model

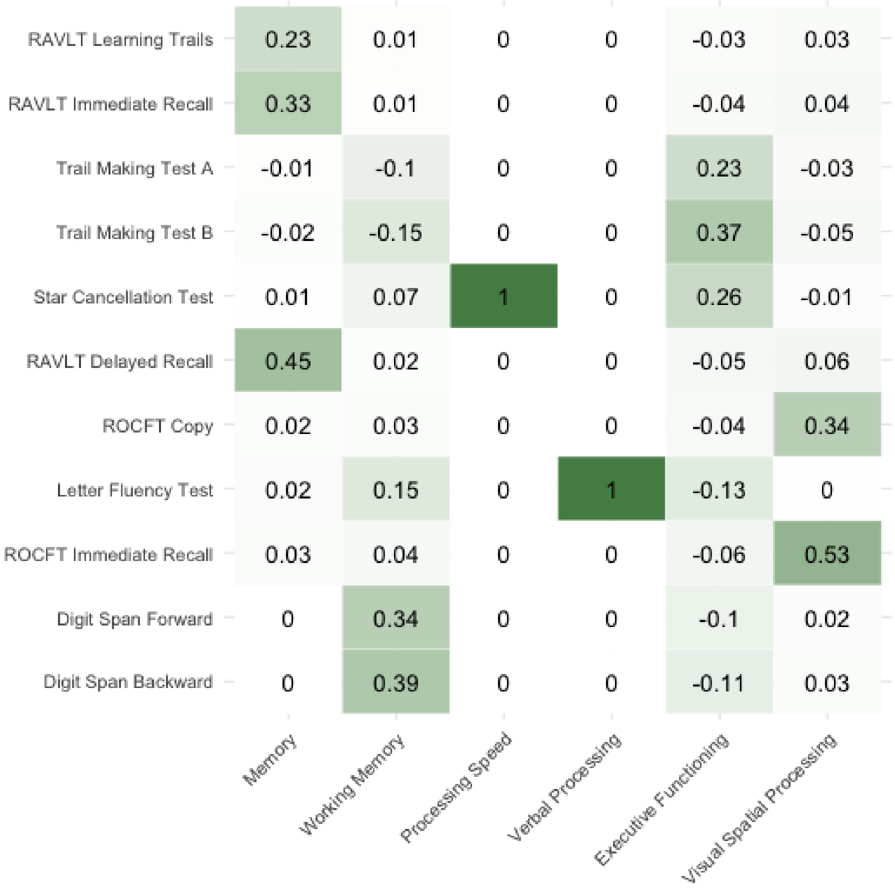

*Figure **S1** legend: Graphical representation of confirmatory factor model. Values have been estimated in a US population 687 of healthy adults. These weights have been applied to the norm-adjusted test values to estimate the cognitive z-scores. Note for Processing Speed and Verbal Processing, only a single test determines the normalized cognitive z-score.*

The weights for the cognitive z-scores have been estimated by fitting a confirmatory factor analysis in the entire cohort of 687 healthy individuals (see Supplementary Figure 2). The structure of the factor model was pre-defined as follows and implemented with the lavaan package in R.

Memory = RAVLT Learning Trials + RAVLT Immediate Recall + RAVLT Delayed Recall
Working Memory = Digit Span Forward + Digit Span Backward
Processing Speed = Star Cancellation Test
Verbal Processing = Letter Fluency Test
Executive Functioning = Trail Making Test A + Trail Making Test B
Visual Spatial Processing = ROCFT Copy + ROCFT Immediate Recall
